# 496. Epidemiology, virulence and antimicrobial resistance of hypervirulent *Klebsiella pneumoniae* and *K. pneumoniae* complex member in western New York, 2017-2020

**DOI:** 10.1093/ofid/ofac492.554

**Published:** 2022-12-15

**Authors:** Brenda L Tesini, Samantha Taffner, Andrew Cameron, Nicole Pecora

**Affiliations:** University of Rochester, Rocheter, New York; Brigham & Women's Hospital, Boston, Massachusetts; University of Rochester, Rocheter, New York; Brigham & Women's Hospital, Boston, Massachusetts

## Abstract

**Background:**

Klebsiella pneumoniae is a leading cause of nosocomial infections, and hypervirulent K. pneumoniae (hvKp) is an increasingly recognized community-associated pathogen. However, the epidemiology and pathogenicity of hvKp and K. pneumoniae complex infections in the US remains poorly understood. We characterized the tissue tropism, antimicrobial resistance, and virulence factors associated with K. pneumoniae complex members and hvKp using whole genome sequencing.

**Methods:**

We analyzed all Klebsiella pneumoniae isolates from liver sources from 2017-2020 and isolates from other sterile tissue sites from 2018-2019 in a western New York regional academic microbiology laboratory by whole genome sequencing. Only one isolate per patient was included. Basic clinical and demographic data was obtained from the medical chart. Virulence factors and antibitoic resistance genes were identified using Kleborate version 2.0. Hypervirulence was defined as the presence of rmpA or rmpA2 and the aerobactin gene cluster (iuc).

**Results:**

We sequenced 25 Klebsiella pneumoniae isolates from liver sources from 2017-2020 and an additional 29 isolates from other non-blood sterile sites from 2018-2019. 80% of the hepatic K. pneumoniae complex isolates were K. pneumoniae sensu stricto (n=20). The remaining isolates were identified as K. quasipneumoniae (n=3) and K. variicola (n=2). Non-hepatic tissue isolates contained only one K. variicola; the remainder were K. pneumoniae sensu stricto. Seven isolates met criteria for hypervirulence; all were K. pneumoniae sensu stricto isolated from liver abscesses (28% of such isolates). All were community onset infections, and only 2 patients with hvKp had prior international travel identified. None had additional sites of infection documented. Four isolates were identified as ESBL phenotypically and all had blaCTX-M-15 detected; one of which was K. quasipneumoniae.

**Conclusion:**

This study suggests that hypervirulent K. pneumoniae hepatic abscesses may be more prevalent in the US than previously appreciated. Non-sensu stricto K. pneumoniae complex members were infrequently isolated from bodily tissues, less likely to carry hypervirulence genes but may harbor extended antibiotic resistance.

**Disclosures:**

**All Authors**: No reported disclosures.

